# Public Actors Without Public Values: Legitimacy, Domination and the Regulation of the Technology Sector

**DOI:** 10.1007/s13347-020-00441-4

**Published:** 2021-01-20

**Authors:** Linnet Taylor

**Affiliations:** TILT, Tilburg University, Tilburg, The Netherlands

**Keywords:** Big data, Political legitimacy, Social media, Governance, Public sphere

## Abstract

The scale and asymmetry of commercial technology firms’ power over people through data, combined with the increasing involvement of the private sector in public governance, means that increasingly, people do not have the ability to opt out of engaging with technology firms. At the same time, those firms are increasingly intervening on the population level in ways that have implications for social and political life. This creates the potential for power relations of domination, and demands that we decide what constitutes the legitimacy to act on the public. Business ethics and private law are not designed to answer these questions, which are primarily political. If people have lost the right to disengage with commercial technologies, we may need to hold the companies that offer them to the same standards to which we hold the public sector. This paper first defines the problem and demonstrates that it is significant and widespread, and then argues for the development of an overarching normative framework for what constitutes non-domination with regard to digital technologies. Such a framework must involve a nuanced idea of political power and accountability that can respond not only to the legality of corporate behaviour, but to its legitimacy.

## Introduction: the Problem with Data Governance

Datafication poses serious challenges to the way people conceptualise rights and freedoms around the world. Privacy, justice, fairness and other fundamental values are being put under pressure by our digitising societies, and much of this process is private-sector led. The economic power of technology giants has for some time been comparable to that of states (Broeders and Taylor 2017), and their political power is rising to match. This technological power has also become an essential component of the state’s ability to govern: people’s ability to opt out of using digital technologies for many of the basic functions of citizenship and public life is diminishing as countries progress from promoting e-government to depending on commercial technology as infrastructure for emergency response, national security, border control, education, law enforcement, and many other public needs (Prins et al. 2012).

This technological entanglement of the private with the public brings the global technology sector immense political power and with it the potential for domination (Laborde and Ronzoni 2016; van der Sloot 2018). The political tools we have developed for controlling this kind of power have not so far been brought to bear on the private sector, and so far are offering little protection from the population-level influence they generate in the public and political spheres.

Problems occurring as a result of this kind of domination can be typologised as follows. First, there are problems arising from contracting. These are cases where commercial firms develop capacity that is complementary to that of the state and then become incorporated as contractors in the state’s operations while still retaining a private-sector identity. One example of the problems this can create is the interaction between the South African government and the firm Cash Paymaster Services, owned by the technology firm Net1, for the digital distribution of welfare payments (Foley and Swilling 2018). CPS became a monopoly provider due to its technical capacity in electronic transfers and biometric verification, but then exploited both its monopoly and grant recipients’ poverty by using the data it gathered through service provision to create a private marketplace in which Net1’s other subsidiaries could market products and services, making deductions directly from welfare recipients’ welfare accounts. When CPS was challenged in court and proved to have engaged in corrupt practices, however, it did not lose its contract due to the necessity of its public function. Foley and Swilling note ‘the risks which come with the outsourcing of services to the private sector […] – where a private company attempts to hold the country’s most vulnerable people hostage and leaving the government with no option but to concede to its demands.’ This is an example of technology-based power that aligns with the three characteristics of domination according to Lovett (2010): imbalance of power, dependency and arbitrariness. These are particularly relevant because welfare recipients cannot say no to the services of a government contractor.

The second way in which technology firms acquire mass influence and leverage over public attention and engagement is in the style of the multinational technology giants: independently of government, through people’s engagement with their platforms or services. This mass engagement effectively makes them public service providers, because their platforms or services can be used to reach particular populations for political, economic or social purposes. An example of this is the API service developed in 2020 by Google and Apple to facilitate contact tracing during the Covid-19 pandemic, where the two firms’ almost complete monopoly on smartphone operating systems enabled them to channel information about the location of, and contacts between, almost all the world’s smartphone users to anyone building an app. A second illustration of this problem is the trend amongst younger US adults toward accessing political news mainly on social media (Pew 2020), giving social media firms’ algorithms immense power over the topics people become aware of, and how they are presented. A third was Facebook’s 2019 proposal to introduce a new digital currency, Libra, that would operate worldwide based on private-sector networks and backed up by capital reserves held by private firms. France’s finance minister responded that ‘the monetary sovereignty of countries is at stake from a possible privatisation of money … by a sole actor with more than 2 billion users on the planet.’ (Guardian 2019b). This type of activity by firms demonstrates problems of power, dependence and arbitrariness: firms not only have the ability to act arbitrarily on the mass scale themselves, but create markets for data and profiles that enable others to act arbitrarily as well, without scrutiny or accountability. This second type also takes the form of passive intervention resulting from the design of products and platforms, as for instance when social media algorithms amplify particular political messages over others in a systematic way, or change the reach and power of misinformation about public health issues (e.g. Guardian 2019c).

The third type that can be distinguished is a hybrid one where government allows or invites technology firms to operate in public space but explicitly refuses to regulate their activities. Two examples are the UK government’s interaction with Amazon in 2019, and Alphabet subsidiary Sidewalk Labs’ marketing of digital payment and logistics platforms to cities around the world. In the UK case, the government agreed in 2019 to give Amazon access to National Health Service data because, according to Health Secretary Matt Hancock, ‘allow[ing] Amazon Alexa devices to offer expert health advice to users would reduce pressure on “[…] hard-working GPs and pharmacists”’ (Guardian 2019a). Under its agreement with the government, Amazon is permitted to monetise copyright NHS content, data and materials by using them to create new products and services, and to share the information with third parties who may also monetise them, but the NHS receives no compensation and has no control over the use of its materials by Amazon (Guardian 2019a). Sidewalk Labs provides digital services to cities in return for all the data stemming from people’s use of public transport infrastructure, which then enable it to capture more of the service provision market as well as channelling demand to its preferred partners, such as Uber (Bliss 2019). Other examples are the World Health Organisation’s adoption of WhatsApp for global public health messaging relating to the Covid-19 pandemic (Devex 2020) and the EU’s call for data analytics vendors to create migration statistics from big data sources (Taylor and Meissner 2020).

This hybrid type of engagement starts with a commercial investment in capacity that results in a monopoly on data about core public services or entitlements. In turn, that data serves to enable companies to capture new markets and create new products that lead to lucrative government contracts. This dynamic is also common to commercial cloud service provision (Gürses and van Hoboken 2017), which started as a byproduct of the surplus computing capacity beyond that necessary for firms’ operations and has resulted in large-scale government contracts for those with unused processing power in their server farms. A 2019 investigation by activist group Mijente found Amazon Web Services had 204 federal authorisations to maintain government data, followed by Microsoft with 150, Salesforce with 31, and Google with 27 (Mijente, National Immigration Project, and Immigrant Defense Project 2019, p. 5).

These problems cannot be remedied within a vision of technology governance that draws a hard line between commercial and public values and activities. Instead, they raise questions of exploitation and power on a more general level. I will argue that it is specifically the potential for domination that surfaces in relation to such scandals, that raises the public interest and that requires a new kind of regulatory and political response, namely one that takes into account the presence of technology corporations as well as states in the public sphere.

So far, responses to informational capitalism as a governance problem (Castells 2008; Cohen 2017) have taken the form of data protection and the developing field of data ethics, which have been demonstrated to have limits in their freedom to problematise and interrogate the political status quo of informational capitalism (Wagner 2018,Taylor and Dencik 2020). I will argue that if we frame the problem of regulating technological power as one of non-domination, the notion of legitimacy becomes a tool to evaluate the extent to which we wish to regulate that power, and how to approach such regulation. Theories of domination, however, have so far been used to analyse state rather than corporate behaviour as a threat to freedom (see, for example, Laborde and Ronzoni 2016; Pettit 2012), despite their utility for thinking about private-sector action. A notable exception is van der Sloot (2018) who examines how the European Court of Human Rights’ decisions on digital surveillance cases have shifted from a focus on interference and harm to a broader logic of domination and arbitrary power.

This paper aims at two objectives: more closely defining the problem of legitimacy in relation to the technology sector, and exploring the potential of a non-domination lens as a way to interrogate the legitimacy of corporate technological intervention.

## What Is Missing from the Current Regulatory Perspective on Data?

### Understanding of the Public-Private Overlap—What Has Changed?

With the rise of the platform and mobile data economies, states worldwide have increasingly come to rely on private-sector data to ‘see’ their populations (Scott 1998), and as a result, the private sector is systematically taking on tasks that have traditionally been the preserve of the state. This reorganisation of activities and responsibilities with regard to digital data is important not just from the legal or administrative perspective but because of its political and social effects.

In this paper, I therefore distinguish between the *public sector*—a functional definition denoting actors and institutions whose activities take place primarily under the auspices of government—and the *public sphere*, where public functions are performed and matters of public concern are dealt with. In relation to entrepreneurial action with technology, the public sphere has two important characteristics: first, it is the space where ‘general’ or ‘population-level’ action can take place—in this case, data-enabled interventions that potentially affect most, or all people in a country. Second, it is the space where interventions can be experienced as social and political, and where there is the possibility of collective understanding and responses. This usage derives broadly from the work of Dewey (1991 (1927)) and will be discussed further below.

It matters how technology firms engage with public-sector systems, not just for the formal reason that in most of the world the private and public sector are subject to different kinds of regulatory scrutiny. Colona and Jaffe (2016) demonstrate that where hybrid governance arrangements develop, they shape what the state perceives as acceptable over the longer term. The interaction between Chinese state authorities and mobile network operators during the Covid-19 pandemic in 2020 for example resulted in various tools for social control which rapidly became part of the apparatus of everyday Chinese governmental power (The Guardian 2020). This suggests that the worldwide shifting of public-sector tasks such as public health and law enforcement onto private-sector actors therefore requires a re-ordering of democratic and regulatory scrutiny.

Establishing meaningful accountability for the private sector is a particular challenge in relation to technology. This is partly due to the opacity of many large-scale technical systems, but also because governments often make an economic argument that such firms should be protected from public scrutiny. For example, in relation to the NHS-Amazon data sharing deal, the UK’s Department of Health claimed that the contract could not be made public because this might ‘prejudice the commercial interests of Amazon’ (Privacy International 2019). The statement went on to explain that

‘The public interest in the disclosure of the agreement with Amazon is largely focussed on the issue of sharing personal data. The redacted clauses in the agreement cover unrelated commercial issues and therefore do not advance the public understanding of the issue of sharing personal data.’ (ibid)

The Department of Health is making two related claims: first, that Amazon’s commercial interest supersedes the public interest, and second, that the public interest can be defined narrowly in terms of data protection. If no identifiable personal data is being shared, the UK government claims, then the public may not question the right of Amazon to make a profit from their public health service, despite the fact that the NHS is designed to create public goods, and is sustained entirely by taxation.

How does this kind of public action by technology firms differ from the public-private partnerships that have become a normal mode of public service provision in most higher-income countries? This is not merely an alliance that benefits government and firms, nor is it just another example of the neoliberal state exercising the principles of New Public Management. Instead, a different type of claim is being made by both parties involved: that there is no difference between public services provided by government and by business, despite the profit interests involved and the different regulatory architectures occupied by firms and government. In the classic PPP model, firms contract with government in sectors where they already have expertise, with engineering firms for example contracting to build public infrastructure, and software companies developing platforms to provide e-government services. In contrast, this new mode of involvement is based on formerly unrelated capacity (data processing, cloud storage, analytics) entrepreneurially repurposed to take on public service tasks in a particular field, but without the ‘implicit values, norms and skills’ that characterise existing actors in that field (Sharon 2020: 7). Sharon uses both Walzer’s concept of spheres of justice and the idea of political legitimacy to explain why this new phenomenon is problematic in the case of Google and Apple’s contact-tracing API: ‘legitimate advantage acquired in the sphere of digital goods— digital expertise—has been converted into advantages in the sphere of health and medicine (where epidemiological expertise should be the main source of legitimacy), and in the sphere of politics (where democratic accountability should be the source of legitimacy)’ (Sharon 2020: 10).

As well as the transgression of spheres of expertise and values, such forced marriages between private and public also undermine transparency and contestability. While a lack of public transparency is normal for business contracts, this becomes a problem when firms undertake the tasks of government. When the EU instigated a market for migration statistics deriving from big data (EU 2018), vendors rushed in with projects to track and predict migrants’ movements and behaviour, with an eventual audience of the IOM, Frontex and other border and migration authorities. However, this contribution to migration policy was just as opaque to the public as the work of Google and Amazon with public data. A freedom-of-information request to Frontex in 2019, asking to see documents relating to the Big Data for Migration projects, resulted in a reply from the organisation’s Transparency Office that

‘Frontex has identified a total of 28 documents. However, access to 27 of those must be refused […] as their disclosure would undermine the protection of commercial interests of legal persons. […] As no overriding public interest that is objective and general in nature and not distinguishable from individual or private interests for the release of these documents is ascertainable in the present case, the release of these documents has to be refused.’^1^

Similarly to the UK’s statement about Amazon, this is a claim that private firms’ activities on public issues must not be defined as public in nature and subjected to public scrutiny. This claim twists the purpose of data protection, which was originally conceptualised as a way of protecting democratic rights, regardless of whether states, firms or both were threatening them (Westin 1967, p. 65). In public administration terms, where firms formally contract with government, this means that responsibility and accountability for service provision formally remain with the state, but research shows that in practice, this tends to be a rhetorical rather than an operational claim since such functions are not usually highly visible in terms of procurement and execution (Mulgan 2006).

### Defining What Is Public Technology

Even where they have widespread negative effects on political, social or economic life, firms tend to be judged according to different standards from government. This is because firms ‘do not function according to a democratic logic to offset inequalities or to defend certain rights’ (Elsig and Amalric 2008) unless they are explicitly forced by government to do so, and moreover because regulation of the private sector is not designed with public scrutiny in mind. The functional and processual ways of considering legitimacy common in public administration scholarship, i.e. those of input, output and throughput legitimacy (Scharpf 2009), are not designed to help think through the role of firms when they act on the public at scale. Technology firms’ new role fits imperfectly with the theory available, so that the latter needs adapting to provide traction on private actors acting in public ways.

One place to start is by locating and defining risk in relation to large-scale technological interventions. What should be the target of transparency claims—personal data, or an intervention per se? French administrative law offers one possible approach, as demonstrated in the 2016 ‘Loi pour une Republique Numerique’. The law names a category of ‘Données d’intérêt général’ (data of public interest) separately from the national law on data protection, and stipulates that this category of data must be opened for public use and the firm appropriately compensated. Although the French law largely assumes this kind of data will be created through public-private partnerships, it may open the door to claims to other kinds of data as ‘public’. This assumption that data becomes a matter for the public and the government (Art. 17),^2^ rather than the government and the firm, contrasts explicitly with the UK government’s position on its Amazon Alexa collaboration, i.e. that a tech firm is entitled to make profits from public data without being accountable to the public.

### Updating Corporate Legitimacy for the Technology Domain

I have argued above that the constellation of public-private, private and hybrid technology interventions described above is not simply a new permutation of the public-private partnership. Instead, technology firms are behaving as autonomous actors with (in relation to their specific fields of operation) some of the reach and power of government, and this poses particular and new risks. These include the following:

#### The Scale and Reach of Corporate Technological Power

The scale and asymmetry of power and information with regard to data technologies—namely, how much data is being collected and used, the invisibility of that process, and the scale and depth with which it may be used to intervene on people—has exponentially grown over the last decade. The scale of corporate power over people through data, combined with the ubiquity of the private sector in the public sphere, means that increasingly people do not have the ability to opt out of providing their data to firms—and that states have little option but to turn to technology providers for data on the public, as could be seen with the Google/Apple API developed in the Covid-19 emergency.

#### Technological Shaping of Operations of Citizenship

The ways in which populations can be influenced and manipulated through the data they emit have developed exponentially over the last decades. Corporations project themselves into the public sphere in ways that have the potential to render democracies fragile and to empower the private sector at the expense of the state—through establishing independent currencies, intervening in electoral politics, determining which migrants can be seen and which remain invisible, who goes to jail and who stays free. These interventions have implications for civil and political rights. Regulation is equipped to counter concrete and visible harms such as false representation, unequal treatment, service denial due to inaccurate data and leakages of personal data. Instead, however, the involvement of big tech on the societal level creates risks relating to distributed visibility, to chilling effects on speech and action, to problems of autonomy and generally to slower-moving, multidimensional problems.

#### Technology’s Tendency to Create and Amplify Vulnerability

The AI phase of datafication not only brings new types of harm, it also inflicts it unequally. Eubanks (2018) has explored the ways in which the automation of public service provision disempowers the poor and vulnerable by removing the personal knowledge and empathy of social workers from the bureaucratic environment, by ensuring that errors persist and are passed along the bureaucratic chain of care provision, and by making it harder for people to correct errors where they are identified. Kulynych et al. (2020) look at the ways in which algorithm-based optimisation processes disadvantage non-users of technology in the name of participation (those who use the right devices to communicate their needs become visible at the expense of those who do not), for example how Uber’s optimisation decreases support for public transport systems, which in the US affects the mainly lower-income people who rely on them.

As well as exacerbating inequality, intervention by commercial firms can create new vulnerability. CPS’s welfare grant distribution contract in South Africa imposed both the English language and digital systems on recipients who were unused to both, marginalising them in new ways and then taking advantage of that marginalisation as a market for goods and services. Similarly, experimentation with blockchain and self-sovereign identity projects (ID2020 2019) by technology vendors on refugees and displaced people create guinea-pig populations who can both be exploited as new markets and serve as proof-of-concept for market expansion plans elsewhere.

#### Lack of Global, Accessible Frameworks for Effective Redress

When commercial systems handle public-sector functions, particularly across national borders, the link between citizen and authorities is frequently broken. If a Syrian refugee placed in Lebanon decides that they want to defend themselves from the possible negative effects of data analytics by the World Food Program (Responsible Data 2019), they will need to first identify what is being done with their data by a US partner, Palantir, then make a claim either against that US firm or against the WFP, a humanitarian body which is legally immune (Boon 2016). To do either, they will need to go through the Lebanese state, whose data protection law is designed to promote commerce rather than protect people (SMEX 2018). These problems are part of the larger structural impunity created by globalisation: as Nancy Fraser points out in her work on ‘abnormal justice’, ‘in the wake of transnationalized production, globalized finance, and neoliberal trade and investment regimes, redistribution claims increasingly trespass the bounds of state-centered grammars and arenas of argument’ (Fraser 2008, p. 396).

## Interrogating Legitimacy as a Route to Non-domination

As argued above, when technology corporations intervene on the public they do so largely outside the normative and legal frameworks that mediate the power of state authorities over citizens. If the technology sector continues to evolve toward state-like functions, this suggests it may need to exist within a more state-like framework. Given that firms exhibit different levels of state-like behaviour and potential for political domination, this suggests that regulation should be shaped according to how state-like and potentially domination-related their actions are in relation to the public.

Although data protection is usually cited as the key to controlling the power of technology firms, its underlying premise—the rational, informed, liberal subject—is vulnerable with respect to the new paradigm of big data and AI. In this new paradigm, what happens to data after the individual (often unconsciously) generates it is largely opaque and takes place mainly within corporate architectures. The terms-of-service information people receive about data’s lifecycle usually refer to ‘research’ and ‘third parties’ as if this constituted meaningful information based on which people could exercise rights over their data. Everyday life in the data economy, however, demonstrates that this kind of control and knowledge are an illusion. Moreover, many of the forms of data that have the most impact on our lives are inferred or created as derivatives from data we are aware of, and circulate beyond the reach of our individual rights (Wachter and Mittelstadt 2019).

While data protection relies heavily on the idea of the ‘legitimate use’ of data, there has been little debate on its conceptual underpinning. Europe’s GDPR, for example, cites the idea of ‘legitimate purpose’ as if it were self-evident where the legitimacy to act using data is sourced. It does not provide guidance on where we should seek the definition or criteria for what is legitimate and what is not. In the US and other jurisdictions, there is even less clarity on what constitutes legitimacy. There, data protection provisions tend to be sector-specific and to address people as consumers rather than citizens, which leads to framing legitimacy in relation to commercial, rather than public, interests. If instead we demanded that government take responsibility for technology firms’ engagement in public functions, the state would find itself in a position similar to the one it holds in a public-private partnership, and individuals would not be in the position of having to regulate firms’ actions without the necessary power. Instead, it would become possible for watchdogs and regulators to push the state to create conditions of transparency and to limit firms’ engagement to avoid function creep.

With regard to public authorities’ own data use, the GDPR is specific (in Art. 6(1)(f)) that they must locate the basis of their ‘legitimate use’ in national law; however, it does not demand this of corporations. This is because the responsibility for making sure corporations are operating within the law lies with the state (see, for example, the Ruggie Principles for human rights and business (Ruggie 2011)). However, this involves no positive obligations, so that if a firm starts taking on the tasks of public authorities, questions arise about the type of legitimacy involved. For example, if Amazon starts to intervene in public healthcare provision or in the insurance market based on its access to public-sector data, it is unclear how people should weigh the legitimacy of those interventions—on the same basis as government, in which case where is the law that allows it to shape public health? Or on the much weaker basis of its business interests, which do not seem sufficient to bound this scale of power?

In a thin interpretation of legitimacy, a firm should just act within its own mission and not disobey the law—as the UK government argues Amazon is doing, for instance. Is this enough though, or do we need to apply a thicker concept of legitimacy so that a corporation that gains the ability to intervene on the public and affect public goods and values also becomes subject to the kinds of legitimacy demands made of the state?

We see an alternate framing of corporate (il)legitimacy from constitutional courts in South Africa and from the Supreme Court in India, where problems of corporate exploitation of the public through data have forced a consideration of firms’ standing in relation to the public. In South Africa’s CPS case, the welfare system became dependent on the technology firm so that even after it was judged to be engaging in corruption and fraud, its contract was renewed to prevent interruption in the delivery of welfare grants.^3^ In formal terms, this legitimised CPS (Foley and Swilling 2018, p. 44) although the court’s and the petitioners’ statements are clear that this was not the objective. This aligns with the Indian Supreme Court’s judgement regarding the possibilities for exploitation inherent in the country’s Aadhaar population database^4^—by the time the case came to judgement the activity of the company had become inseparable from the responsibility of the state to its citizens, influencing the judiciary to leave a system in place, despite agreeing it had the potential for exploitation and domination.

### Thickening the Concept of Corporate Legitimacy

Political philosophy perspectives on legitimacy have centred on the relations between states and their citizens. The domain of business ethics has more recently addressed the question of how corporations can claim legitimacy, at first with a focus on the economic relationship between corporations and society (e.g. Epstein 1972). It is worth briefly engaging with the business literature on empirical legitimacy in order to address the argument that we do not need a normative legitimacy framework for corporations in the public sphere and that instead we can rely on them to determine for themselves the values they will uphold, independently of the public.

In the 2000s, as globalisation accelerated, researchers started to question the idea that corporations could set their own criteria for legitimacy. Palazzo and Scherer (2006) chart how issues such as multinational firms’ labour practices in low-income countries, or the violation of environmental rights, have in the past generated challenges that had to be answered in the political, rather than purely the economic, sphere. Building on this, Demuijnck and Fasterling (2016) posit two categories of corporate legitimacy. First, that a firm is *normatively* legitimate if it can be supposed that people would approve of its entire business model and practices, if they were fully informed of them. Second, that it can be *empirically* considered legitimate if it is popularly perceived to be so. The important factor here is whether people are informed about what the firm is doing: the authors warn that this empirical legitimacy may depend on ‘some information [being] hidden or misrepresented so that people can be manipulated’ (ibid, 678). These paper-thin concepts of legitimacy are particularly unsatisfying because the ways in which technology firms monetise data are hidden from the public. In order to make personal choices on the everyday level with respect to personal autonomy and liberty, people also need meaningful information on the overall business model they are engaging with. This necessitates a thicker concept of legitimacy against which to test business purposes, if we are not to legitimise anything a corporation chooses to put in its mission and then enact on the public.

Classic theories on legitimacy are of limited help here because they deal with different concerns. Firms do not, for instance, (usually) claim coercive political authority, meaning that the coercive power that has been the focus of political philosophies of legitimacy, starting with Rousseau, may not be the most relevant framing when we think of the private sector. Technology firms are not attempting to conscript citizens, to go to war or (directly) to conduct coups. Instead, we see them claiming a passive kind of political legitimacy, namely that they may explore activities that have traditionally been those of the state while remaining shielded from public scrutiny as purely for-profit actors. There is an important difference between this type of activity and government procurement, or the purposeful privatisation of public services and infrastructure. In these cases, it is clear that we should hold the government responsible for the outcomes of privatisation. There is also a difference between firms using open data that is accessible to anyone. The political legitimacy problem belongs to a still-emerging middle ground that allows firms with particular reach and power to become quasi-governmental actors while still claiming to be merely doing business.

The cases where we might want to require a thicker version of legitimacy from firms can be grouped into particular types of function. First, sorting and categorising citizens for purposes of population-level intervention. Second, activities that have large-scale impacts on fundamental rights such as free speech and freedom of association; for example social media platforms which must decide whether to allow misinformation to be publicised or controversial interest groups to be organised in online space. Finally, we might pay attention to firms’ activities that constitute information policy usually undertaken by government (for example on public health, law enforcement or emergency information services).

## Corporate Authority and Public Consent

Debates on the nature and foundations of legitimacy take two directions. One, originating with Weber (1918), is based on the notion of effective authority, i.e. that an actor is judged legitimate if people accept its authority and agree they should obey its commands. An alternate perspective on legitimacy holds that an actor may be judged legitimate if its power is understood by people as justified; Raz, in particular, links this type of legitimacy to the justification of authority (Raz 1986). According to him, people may obey an actor for one of two reasons: either because they have effective authority (for example, a first-aider asking people to stand back when someone is taken ill in the street), or because their authority is based on legitimacy and therefore need not be weighed in the same way (for example, a firefighter asking people to leave their homes because their neighbourhood is at risk). The first case does not preclude disagreement and resistance: for example, if I were a doctor, I might not want to stand back but instead step in and take over authority. In the second case, I would be unlikely to refuse to leave my house regardless of my opinion of the level of risk involved.

The distinction between these two types of authority is important to the issue of corporations in the public sphere because it enables us to ask what kind of authority is being exercised or obeyed. Should we address Facebook, for instance, as a political actor claiming the legitimate authority to allow or disallow particular kinds of speech, or as a business with merely effective control over its users’ behaviour? It matters which we choose because they lead to different routes of action, the first in the political sphere and the second in the sphere of regulation, for example through data protection or competition law. It is hard, however, to argue solely for the latter, and practice seems to bear this out: although data protection and competition law are clearly the main practical tools available if we wish to limit the firm’s power, its management is increasingly being called to account by state authorities because of its political effects.

It matters to this discussion what kind of political effects a firm is generating. In the case of privatisation, political effects can effectively be channelled back to the government: if, for example, a rail provider runs the trains late or dangerously, this will have political repercussions for the ministry responsible for privatising the network while the firm itself will experience primarily regulatory repercussions with the aim of restoring service to the public. In contrast when technology firms create new systems or applications, rather than contracting to serve a specific public need, they are carving out possibilities for engagement and profit in the public realm. This raises the question of how the public should and can engage with any problems firms’ actions create: indirectly through government in the form of regulation and law, or directly through cooperation or its withdrawal.

If we wish to find a thick enough conceptualisation of firms as political actors, and the relation of public consent to that role, we may look to contractarian philosophers such as Hampton, whose definition of morally legitimate political authority distinguishes between ‘convention consent’ where people may cooperate with an authority even though they find it unjust because they see no reasonable alternative to it, and ‘endorsement consent’ where people agree with the authority on a moral basis and therefore find cooperation justified (Hampton 1997, pp. 100, 112). This distinction can also be found in Rawls’ articulation of ‘joining consent’ as opposed to ‘originating consent’ (Rawls 2008, p. 124).

The notion of consent, and the kind of relationship within which consent can take place, is relevant because of the frequent misuse of the notion of consent in relation to technology. With the South African example of CPS as welfare distributor, for example, welfare claimants became ‘users’ and had to give consent to the firm to use their data, a process that effectively replaces the consent of the citizen to be governed by the state and to receive entitlements as part of that relationship. In this case, imposing the hollowed-out version of consent demanded of users of commercial technology is the epitome of forced cooperation. Evidence of this hollowing-out of consent is provided by Draper and Turow’s research on people’s relationship with technology firms in the USA: the authors find that people believe ‘available responses are meaningless in the face of various manifestations of corporate power’, and that this ‘prevents individual frustration from being transformed into collective anger that might encourage institutional change’ (Draper and Turow 2019, pp. 1834–5).

We can connect the kind of hollowing-out of consent that occurs in the realm of commercial technology with the hollowed-out political consent Hampton warns is problematic because it is used as a way of coopting resistance and preserving the status quo:

‘[the state] allows people to withdraw their consent, at regular intervals, from particular persons holding power and particular rules or offices in the regime, even while keeping them within the overall political structure of the regime. It is therefore a system of political authority that attempts to maximize the convention consent it receives from residents of a territory by providing politically acceptable avenues for those residents to rebel against aspects of its operation.’ (Hampton 1997, p. 107)

We can see this in action if we look at privacy campaigner Max Schrems’ 2015 victory against Facebook. Schrems claimed the ‘Safe Harbour’ law allowing companies to transfer data about EU citizens to the US, and in turn to US intelligence authorities, violated his fundamental right to privacy. The Court of Justice of the European Union found in his favour^5^ and struck down the law. It was, however, replaced by a similar law, ‘Privacy Shield’, which then proved to be merely a figleaf under which identically illegal transfers could continue (Guardian 2020).

On one hand, evidence of meaningful consent would be important to consider. Before we assume that we need to guard ourselves from domination by technology firms, we should check the arguments to the contrary. What if technology firms are justified in arguing that their contribution to the public sphere outweighs any problems they cause? Given how readily states are giving up their power to corporations, and those corporations’ strong arguments for their contribution to the public good (Taylor 2016), we should consider this too. If this narrative is the right one, then we might expect it to be generating evidence of real consent. What endorsement consent might look like in relation to corporate activities remains an open question however, given the evidence presented by Draper and Turow that actively using a firm’s products or services may denote unhappy resignation rather than agreement with its business model. On the other hand, if convention consent is what we currently see with regard to the role of technology firms in the public sphere, and if contestation within the existing framework is not a meaningful option since states are protecting the interests of corporations as their own, this suggests that domination is an appropriate framing for the problem of technological power.

The problem of domination has long been discussed both by liberal philosophers and in critiques of globalisation. Laborde and Ronzoni (2016, p. 279) argue that the power of multinational corporations inevitably gives rise to domination—which they define as ‘subjection to the arbitrary power of another actor’—on the state level. These corporations’ power makes them rule-makers and the states where they operate rule-takers, decreasing public control over them. The authors describe how ‘transnational private actors […] exercise arbitrary power over crucial aspects of domestic institutional settings’ and ‘not only do domestic institutions have limited means to resist the phenomenon; they are also often themselves pressurised into modifying their own policies, thus being undermined both in their problem-solving capacity and in their accountability to their own citizens.’ (ibid, p. 282). This seems, on the whole, a fair description of the current state of the technology sector as a global actor in the various fields where it operates.

### The (Technologically Mediated) Sphere of Public Reason

If we wish to understand the problem of domination, and why user consent is not an answer to it, we must ask whether disengagement is possible. The large-scale involvement of the private sector in datafication challenges assumptions about the link between what is population-wide and what is public. Dewey (1991) writes that any policy-scale intervention has the side-effect of creating a public who can then contest it. The view of Lukes (2004 (1974), p. 20) adds nuance to this by arguing that issues have to acquire a specific public in order to be dealt with, and if they do not, they will not be addressed. To use Lukes’ logic, if businesses are shaping and even initiating policy interventions, but the technology used to do so has already been introduced and normalised as something innocuous (such as a social network or a digital assistant, for example), it may be harder for new uses of that technology to become perceived as problematic and thus to become an issue that acquires a public to debate it.

This question is important given that technology firms mediate exactly the processes of public reasoning that are usually understood to characterise legitimate action. As it becomes less possible to disengage from certain technologies, this creates a monopoly problem where the public can only see the world as it is presented by particular actors. This is not a new problem: Dewey identified it in the 1920s when he observed that

‘Industry and inventions in technology, for example, create means which alter the modes of associative behavior and which radically change the quantity, character and place of impact of their indirect consequences. […] These changes are extrinsic to political forms, which, once established, persist of their own momentum. The new public which is generated remains inchoate, unorganized, because it cannot use inherited political agencies. The latter, if elaborate and well institutionalized, obstruct the organization of the new public. … To form itself, the public has to break existing forms (Dewey 1991 [1927]: 30–31).

This problem of ‘inherited political agencies’ evokes the questions raised by large-scale influence over public discourse on the part of firms. When political communication is mediated by commercial algorithms, our understanding of the meaning and effects of the incursions of technology firms into the public sphere is also inevitably shaped by them, just as Dewey describes.

Scholars such as van Dijck (2012) and Helberger (2019) ask how we should regulate the algorithmically mediated public sphere and what criteria we should use to evaluate whether active participation in society is still possible in the presence of commercial interventions. Should they be addressed as a political and economic phenomenon that must be shaped in the offline political domain, or should we instead engage with the technology through techno-regulation to shape its effects? Which would produce ‘more accountable relations’ between the powerful who use data and those upon whom it is used? (Daigle and Ramírez 2019) Even if we find a way to reconcile these two approaches, it still matters what we believe ‘the public sphere’ to be. If no one can perceive it other than through the prism of data technologies, we should take into account that this intermediation has a short-circuiting effect on people’s ability to decide what kind of consent, if any, they might give to the presence of these firms in the public sphere.

Dewey argued in 1927 that the body politic had to remain the answer to this problem. Policy interventions, he pointed out, are seldom clearly visible to those directly affected, and ‘[since the] supervision and regulation [of these consequences] cannot be effected by the primary groupings themselves[…] consequently special agencies and measures must be formed if they are to be attended to’ (Dewey 1991 [1927], pp. 15–16). A public must make sense of new interventions and respond to those conducting them, regardless of whether individuals can perceive them clearly or not. Dewey’s ‘special agencies and measures’ are the question here: what would it take for a public to be able to form around, understand and respond to data-enabled harms and risks on the collective level?

Fraser (1990, p. 66) helps answer this question by orienting us toward more inclusive representation that takes account of ‘subaltern counter-publics’. This notion of multiple publics is important in understanding how the social contract with regard to data currently works. Rather than a single ‘we’ who volunteer our data for purposes of governance or commerce, in reality that transaction may be consensual and reciprocal for some groups (for instance in relations between elites and government) and simultaneously oppressive and intolerable for others within the same society (for example indigenous groups, the incarcerated, or welfare recipients’ relationships with those who categorise and sort them through data technologies). In the South African case of CPS, for example, while some welfare recipients may be well served by digitising provision others will have language difficulties with services provided in English and without adequate user support. If those groups become represented, the ability of CPS to serve the public adequately becomes contestable and with it, the decision to move service provision to a private online provider.

This fuller engagement with the public may provide a route to greater legitimacy, but does not solve the problem of domination. Not all publics are created equal, and many of the people who can best identify problems of domination with regard to data technologies are in situations of structural exclusion that cannot be solved by efforts at inclusion. One high-profile example of such a group, explored in the next section, is the subjects of humanitarian action, who are increasingly a population of interest and experimentation for technology firms.

### Case Example: Humanitarian Data

The humanitarian domain presents a hard case for understanding the legitimacy of datafied intervention by businesses for two reasons. First, because international humanitarian organisations have privileges and immunities that insulate them from most legal challenges (Boon 2016), making it hard to use legality as a basis for claiming legitimacy. Second, because the populations they assist are excluded from deliberative processes where they can represent their own interests and have them recognised (Fraser 2008). Yet they are increasingly the subjects of technological experiments involving commercial partner organisations who are also involved in mass surveillance (Responsible Data 2019), identity technologies (Newsweek 2019) and other technologies at the public-private intersection.

The humanitarian sector has begun to debate a bioethics-informed approach to data governance that treats the subjects of humanitarian assistance as able to give or withdraw their consent like other populations. Anna Kondakhchyan of the Cash Learning Partnership, which aims to manage the introduction of financial technologies into refugee service provision, asks:

‘how do we turn “informed consent” from a tick box on a screen during registration into a meaningful process, respectful of people’s rights to privacy and protection? How can we ensure that affected populations understand how their data will be used, and are reassured that it won’t be mistreated by the third parties with whom we work? And what should we do if we learn that the third party we have chosen to work with uses programme participant personal information to either sell them unwanted services, or worse still, refuse certain potentially beneficial services to this group?’ (Kondakhchyan 2019)

The last question seems to answer the previous two: if individuals engaging with public technologies are asked to consent to the monetisation of that engagement, and if the humanitarian partner cannot guarantee that its private-sector partner will not behave according to the same principles as it does itself, informed consent is not possible and instead we find hollowed-out consent that should not be taken as conferring legitimacy, and may instead be taken as a signal that the potential for domination is present. This is indeed the case, given that firms partnering with humanitarian organisations remain independent and do not adopt the mission and principles on which the sector’s effective legal immunity is based. On that basis, informed consent on the part of the population is neither relevant nor possible, and other criteria for legitimacy must be sought.

It is this search for legitimacy that characterises the other strand of thinking in the field. In 2018, the Signal Program, a human rights group developing data technologies for the humanitarian field, published the Signal Code (Campo et al. 2018). The Code examines the basis for translating the established legitimating basis of humanitarian organisations into the new partnerships and collaborations occurring around data. Rather than assume that the involvement of data analytics in humanitarian action is a given and asking how to legitimise it, the authors set out in detail the core obligations of the field and the ‘humanitarian standards and related ethical, moral, and legal frameworks’ on which the field’s legitimacy is based. The Code returns continually to the fundamental obligation of humanitarian organisations to answer the needs of affected populations, warning that

‘The use of information and ICTs do not become humanitarian by virtue of their use by humanitarians. For these activities to qualify as humanitarian, they must be designed and executed to uphold the humanitarian principles. An information activity is only humanitarian if its aim is to prevent and alleviate suffering, protect life and health, and ensure respect for the individual.’ (Campo et al. 2018, p. 18)

This framework starts from the position of Raz (1986), namely that an authority gains legitimacy by serving the people. At no point does the Code inquire as to the legitimacy of the technological partners becoming involved in humanitarian action. Instead, the authors focus as comprehensively and forcefully as possible on the requirements and obligations of the field in relation to affected people in order to address what is legitimate and what is not. This effectively makes humanitarian organisations fully responsible for the actions of any for-profit actors in the humanitarian sphere: if they are intervening, the intervention is that of the humanitarian, not of the firm. There is no difference in the requirements of transparency, accountability and ethics regardless of the originator of an intervention: if it is occurring under the auspices of the humanitarian sector, there is one standard.

This case highlights the entanglement between consent and legitimacy in relation to large-scale interventions. If consent is not possible, as it is not where people have no chance to withdraw from the intervention, and if the intervention is all-encompassing for its subjects, then the relationship is inevitably one of domination and in order to minimise it, those conducting that intervention—including technological partners—must demonstrate their legitimacy. If they are not accountable to those they are intervening upon, they require something else, for example a humanitarian mission to justify their actions. A business case is not sufficient to make any claim to legitimacy, particularly in the absence of meaningful accountability. Now that corporations are partnering with humanitarian organisations, there is a debate emerging about whether they can be shielded by those organisations’ unique privileges and immunities given that their mission is profit, not humanitarian action (Responsible Data 2019). There is a similar question for firms doing the work of government: if their mission does not align with that of government, they cannot borrow the government’s legitimacy. Instead, other guarantees of good behaviour, such as public accountability, must correspondingly be ensured.

## (Re)Formulating Accountability for Technology Firms

British politician Tony Benn recommended asking five questions of powerful people or institutions: ‘What power have you got? Where did you get it from? In whose interests do you exercise it? To whom are you accountable? And how can we get rid of you?’ (Benn 2001). His questions were intended as a test for a democratic system, but (following Shaw and Graham (2017)) they are worth translating into questions for technology firms working in the public sphere. If so, they might consist of the following:

1. How does your technology affect people at scale?
2. Are your actions bounded by an articulated purpose?
3. Are you accountable for your effects at scale?
4. Are your contracts and assignments transparent to, and contestable by, the public?
5. Is your involvement in the sector time-limited?

If a firm lacks public accountability for its actions when they impact the public, then all the other questions become irrelevant because the firm, rather than the public, gets to define the answers. Demanding legitimacy of technological power in the public sphere therefore means paying close attention to how the benchmarks and criteria for accountability are set—is a partnership structured so that it can be rolled back if it is demonstrated to be against the public interest? Are risk assessments dealing with a technology’s broader effects accessible by default to public authorities and to the public?^6^ And is accountability channelled toward bureaucracy, where independence from government exists but response may not be seen as urgent, or government, which may be more responsive but also may be involved as a collaborator in the problem?

To return to the domination framing of this problem, multinational firms gain arbitrary power by the sheer extent of data they have access to, which allows them to change their focus at will—Google can transform from a search engine into running urban transport systems; Palantir can pivot from predictive policing to planning aid logistics for 92 million beneficiaries. Scrutinising the political legitimacy of these firms’ activities is one of few effective ways to regulate their accumulation of arbitrary power because the more successful they are at the public intervention, the less possible it becomes for the public to ‘get rid of them’, in Benn’s immortal phrasing, and the less likely it becomes that their engagement will be fixed in its duration. The greater freedom a firm has to do business within a country’s regulatory framework, the shakier its claim to legitimacy becomes.

Two avenues to corporate accountability currently exist. One is that government has the responsibility to keep business within the law, so firms’ accountability could be seen as flowing to some extent through government. The other is the idea that consumer choices should regulate the market, and people should stop using firms which have negative effects on society. However, these two forms of accountability in fact have the effect of blunting each other. Government’s responsibility for firms’ compliance is only activated if they do something illegal, and the scope of this responsibility does not include firms’ inclination to act in the public interest. Consumers’ ability to vote with their feet, while relevant if they are using a business’s platform or services voluntarily, does not apply if that business is acting on them as a member of society. In this case, they are no longer consumers but merely part of the affected population—something that places a different kind of responsibility on businesses not to do harm. This disjuncture in accountability architectures is a serious problem from a legitimacy point of view because it points only at specific and visible actions, and away from firms’ most serious and far-reaching effects on society—the datafied power that allows them to intervene at will in different sectors, and thus risks domination.

However, neither of these mechanisms has so far successfully dammed the power of technology giants. Instead, the perceived risks of new technologies are being answered by incorporating technology firms into governance architectures as decision-makers and standard-setters. The EU’s High-Level Group on AI Ethics involved 52 members, a majority of whom were corporate representatives (AI HLEG 2019), and produced recommendations that lacked any ‘red lines’ for activities where the use of AI was not permissible.^7^ In another example, the UK government’s ‘AI procurement guide’ was designed in 2019 in a process led by the World Economic Forum—a group formed to advance the interests of the world’s largest corporations—with contributions from ‘fellows’ from UK Government’s Office of AI, but also the firms Deloitte, Salesforce and Splunk on an equal basis to the UK government (World Economic Forum 2019). Unsurprisingly, the guidelines resulting from the UK process stress the industry-friendly notion of ‘output-based requirements’, which allow policy problems such as homelessness and public health spending to be framed as analytics problems which benefit from formalisation and optimisation, rather than political ones that require debate and collective input from society (Kulynych et al. 2020).

### Targeting Accountability

Mechanisms for accountability require content. It is hard to escape the recent technical discussions of algorithmic fairness in relation to public-sector work (see, from very different perspectives, Angwin et al. 2016; Monetary Authority of Singapore 2019; UK Government 2019). However, as Keyes et al. (2019) demonstrate, the technology sector has tended to pursue a hollowed-out, thin conception of fairness that lacks moral content as well as social or political context. Although using fairness as one criterion for legitimacy has the potential benefit of forcing a connection between the technical and social debates on the issue (Selbst et al. 2019), it is unlikely that this would end with a meaningful improvement in accountability on the part of government for the actions of technology firms.

Gürses and Dobbe (2020) offer an alternative by centering on the democratic challenge of making private digital infrastructures accountable when they take over and optimise public infrastructural functions:

‘AI frameworks promote reformulating social welfare functions as a problem that can be optimized computationally rather than solved through the complex consideration and negotiation of human experience and expertise, shielding the management of infrastructures from democratic forms of control. How can public interest be assured when it is submitted to these economic terms?’

The authors suggest that instead of looking at individual firms and activities, we use the outsourcing of infrastructure development in particular sectors to ask questions about ‘computational infrastructure’s relationship to the demands of global capital’ and that we tie these questions to states’ democratic accountability for their engagement with the global economic system. Doing this would go beyond demanding accountability for particular actions and instead demand that states answer for their decision to invite in global technology firms as infrastructure-building actors in the first place. This approach assumes that global capital will extract rents wherever it touches down, and that the only source of democratic accountability is between people and the governments that decide to invite it in. Bringing criteria together: a framework for legitimacy.

Legitimacy is a problem of both local and global governance. Without the country level any legal or normative frameworks for action become procedurally unrealistic ways to seek redress on the individual level, or to work against domination on the political level, but without situating some demands and enforcement on the international level, there is no traction possible on multinationals. The challenge overall is to build a basis for what Lindahl (2018, p. 46) terms ‘institutionalised and authoritatively mediated collective action’ that is based in discussions amongst and between existing publics but that can also have effect across national boundaries. It is only by solving this problem that we can arrive at a system for seeking endorsement consent (Hampton 1997) from the public to technological intervention, and that allows us to take meaningful steps to prevent the wielding of arbitrary power by the technology sector.

In order to make it possible for people to give, or withdraw, endorsement consent, we need to first link deliberative processes with bodies designed to mediate and control power over the public, and then bring technology firms under the purview of those bodies. This leads us toward law and regulation, including bodies such as public watchdog organisations, parliamentary committees, national data regulators and sector-specific commissions. Given that the task is to hold states to account for regulating corporate behaviour at a new scale, we are in need of institutions that are independent and robust to economic and political pressure, and that can respond to all the stages of a given application of technology—proposed, planned and operational. The global administrative law movement may hold some answers in relation to the actions of firms, since it espouses the idea that states have to be accountable for, or meaningfully hold accountable, non-state organisations (Kingsbury et al. 2005). Connecting the governance of (technological) corporate action to this agenda may be an appropriate given the international reach and character of many technology firms and the issues they generate. Second, to provide the direction for this kind of regulation, states would have to foster a free and plural debate on what is just with regard to data, including how to balance economic with social imperatives. For this to happen, other types of institution need to be involved—think tanks and activist organisations, academia and funders.

Such an institutional and structural approach needs to be complemented by a strategy of paying attention to the effects of technologies on the public. Pagallo (2017) connects the notion of secondary (i.e. procedural) law to legitimacy with his analysis of the Japanese government’s policy on new technologies such as robotics: marking out experimental zones for scientists and the public to engage with them and to test out whether the controls and boundaries on the science of robotics are empirically adequate. Unforeseen challenges can be addressed with the rules and laws already available, or may be found to necessitate new ones. This combination of attending to procedures for safety and to the public’s response to a new technological intervention can be seen as a form of public discussion and effectively constitutes one type of strategy for determining legitimacy: if robots become perceived as a positive addition to society, they will receive endorsement consent. If not, the experiment can be rolled back.

This procedural approach partially addresses the typology of problems outlined at the start, of public-private, private and hybrid technology interventions. The first—where they contract to provide new digital models for public service provision—might be answered by the deliberation-through-practice approach taken in Japan. So might the third type, the hybrid model of engagement where a firm provides public informational infrastructure on an entrepreneurial basis. In this case, though, this would require engagement by the state in creating deliberative processes that might restrict the actions of the company in question—something states have not so far shown themselves willing to do. The second type of problem, the spontaneous corporate capture of particular functions in the public sphere, is the most intractable because it involves firms acting entirely unilaterally. Instead, this would require this type of capture to be scrutinised systematically by dedicated authorities charged with protecting the public interest, with regular reporting to insert the findings into deliberative processes. This kind of scrutiny might be performed by a coalition of a public watchdog and relevant civil society organisations, but would need to be formalised and funded rather than made the responsibility of activists.

In a legitimacy-based approach to technology regulation, technology may not need not be the primary focus: as Peña Gangadharan and Niklas (2019) argue, the problems of the data economy are not new. Just as the incursion of robotics into social space provokes discussion about employment, healthcare, public safety and education, discussions of other technologies in social space will bring up social and political rather than purely technological or regulatory questions. Economic and informational power asymmetries, issues of autonomy, rights and representation—all these are existing social justice issues that already provoke public response. We have to link ‘technological citizenship’, as the Rathenau Institute terms it (Rathenau Institute 2018), which ‘emancipates the regular citizen in relation to the experts and developers of technology’, with everyday citizenship and with questions of social justice. Many of the challenges attributed to ‘technological citizenship’ involve regulating capitalism, something we already have the tools and experience to do, learned from the challenges of regulating other domains such as financial markets and the energy sector. The challenge is to bring these lessons to bear in the presence of the siren song of innovation and corporate power.

## Conclusions

The current challenges of governing technology demonstrate that data policy is not only economic policy: it is social policy that belongs in the political sphere. We can see this from the failure of efforts to confine data governance to data protection law and data ethics, which have resulted in a model which relies heavily on self-regulation and ad hoc enforcement. During the 2010s, we saw much emphasis being placed on ethics commissions and guidelines as actual regulatory instruments rather than consultative bodies, with the assumption that experts advising on a general level in advance of any application. Implicitly, the current architecture also places huge demands on rights organisations, who find themselves in the role of policing violations without the firm-level access to pre-empt those violations, or the institutional heft to prosecute them. The inefficiency of this model means that in practice most of the burden to report serious violations rests on individual whistleblowers, who then bear the full weight of firms’ response on behalf of society. It is not realistic to expect most people to sacrifice their careers and often their personal safety to fulfil a function that should be pre-emptively performed up by the only actor powerful enough to stop corporations taking harmful action—the state.

The first step in establishing meaningful accountability would be to make government explicitly responsible for what happens to data with effects on the population level, and to establish that no matter who is handling it, they are subject to some form of public scrutiny. This might mean on the country level, for instance, that corporations cannot act on the population without experiencing the kinds of checks and balances that attend state action. In the case of corporate collaborators with international organisations, we may need think of accountability as layered and mutually reinforcing, where the receiving country government and the humanitarian organisation both have to appoint monitoring and enforcement bodies to check each other’s actions on the most vulnerable. This perspective also has implications for the new phenomenon of direct development interventions in lower-income countries by technology corporations (Taylor and Broeders 2015): checks and balances would have to be imposed in the country of the intervention, meaning that institutions would have to be developed which would allow developing countries to check corporate power.

There are several possible objections to the approach proposed here. One relates to its inapplicability in authoritarian contexts where the state itself is not concerned with legitimacy. If the state is not interested in guarding the interests of its people against corporate power, no legitimacy-based approach is possible. There are a minority of states where this would fully apply, however, given that even authoritarian governments are faced with some need to justify their power. Some of the most visible problems of technology in the public sphere occur in authoritarian states, however, with the new power of data technologies only adding to the ability of powerful elites to suppress resistance and exert control. In cases of limited statehood, such as developing democracies, a legitimacy approach has the potential to serve as a political tool for governments to exert power over international interests, as in the example of development interventions by multinationals—but it has little to offer citizens of genuinely authoritarian states. Instead, public resistance must first address the problem of authoritarianism itself.

A second objection might be that individual consent is the best way to avoid exploitation where technology firms engage in large-scale action in relation to the public—and in fact is preferable in a liberal context. This is the argument used in the case of CPS in South Africa described above. However, where technology firms are involved, genuine consent on the basis of understanding the business model and choosing to engage with it inevitably becomes collapsed into the idea of ‘user consent’, a hollowed-out version that has only the most superficial relationship with the political notion of consent and which should not be seen as conferring legitimacy.

A final objection is that corporations already have a responsibility and an interest in behaving ethically, and that they will not survive if they do not. This may be true in the case of neighbourhood firms which are seen and scrutinised by those they serve. But the transnational nature of the technology economy, the immense and largely invisible power conferred by collecting vast quantities of data on the majority of the world’s population, and the intense competition amongst technology giants and resulting pressure from shareholders to grow exponentially, removes any incentive or even ability on the part of large firms to understand the effects of their actions at scale.

A governance architecture based on demanding thick forms of legitimacy holds the promise of demanding democratic innovation. First, it would constitute the application of the precautionary principle where new applications of technology at population scale are concerned. Second, it would demand a very different accountability relationship between government (or international organisations) and corporations, where transparency would become radically more important in contracting. Third, it would require the development of new transparency processes where the government made the nature and workings of corporate engagements with public data more visible to the public, and of new fora for discussion on the national and international levels, providing a stimulus to the growth of international civil society.

An important feature of the governance processes discussed here is that they are dynamic and responsive: they must be able to continually interrogate and respond to the effects of technology within society. Devising ways to interrogate the legitimacy of corporate technological intervention seems a problem of ‘staying with the trouble’ (Haraway 2016) of relating classic theories to contemporary realities of globalisation and transnational practices, and of adapting or rethinking theory for different types of state and social contract. Above all, it means taking into account the new power of technology, and testing the legitimacy of that power against the demands of justice.

## Footnotes

1 Letter from Frontex Transparency Office to The Bureau of Investigative Journalism, London, 14.10.2019.

2 Based on Art. 53-1, **Ordonnance n° 2016-65 du 29 janvier 2016 relative aux contrats de concession**: ‘La mise à disposition ou la publication des données et bases de données fournies par le concessionnaire se fait dans le respect des articles L. 311-5 à L. 311-7 du code des relations entre le public et l’administration.’

3 *Black Sash Trust v Minister of Social Development and Others*, 2017, ZACC 8

4 *Writ Petition (Civil) No. 494 of 2012 & connected matters*, 2018, Supreme Court of India

5 *Maximillian Schrems v Data Protection Commissioner*, 2015.

6 Data protection impact assessments (DPIAs) are not open by default, and are only conveyed to data protection authorities in the case of a high-risk self-classification.

7 A penultimate draft of the AI HLEG guidelines did contain ‘red lines’ for unacceptable uses of AI.
